# Supplementary material for: The interactome of intact mitochondria by cross-linking mass spectrometry provides evidence for coexisting respiratory supercomplexes
Source: Mol Cell Proteomics. 2017 Dec 8;17(2):216–32. doi: 10.1074/mcp.RA117.000470 (PMC5795388; doi:10.1074/mcp.RA117.000470)
Supplement: Supplemental Data [file supp_RA117.000470_133922_0_supp_23978_fzffwf.zip › spectra_annotation/mito_DR_spectra_annotation/248-1-2-1-8-1.pdf]

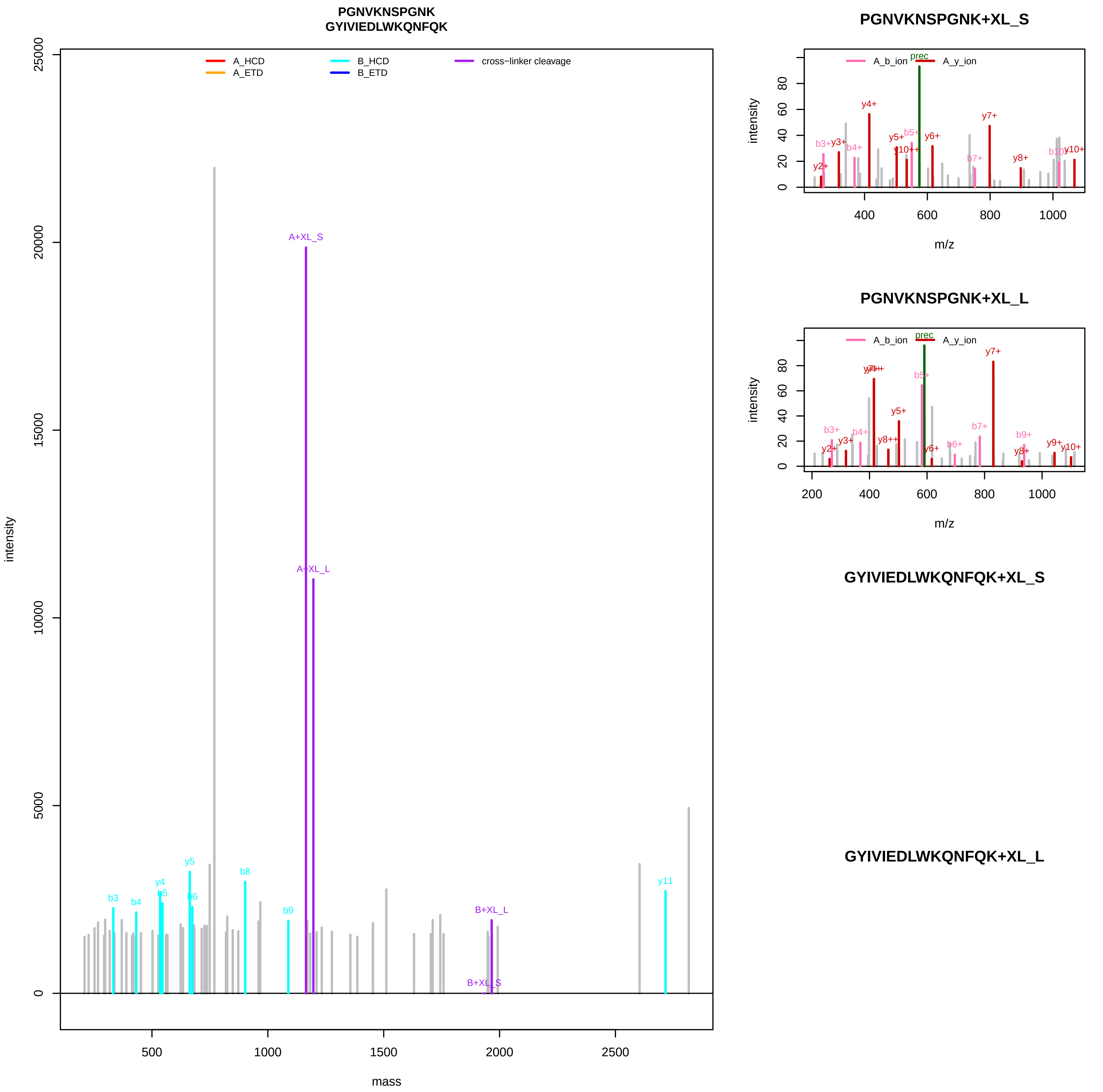

PGNVKNSPGNK
GYIVIEDLWKQNFQK
A_HCD
A_ETD
B_HCD
B_ETD
cross−linker cleavage
A+XL_S
A+XL_L
B+XL_L
B+XL_S
b3
b4
y4
b5
y5
b6
b8
b9
y11
intensity
mass
PGNVKNSPGNK+XL_S
A_b_ion
prec
A_y_ion
y2+
b3+
y3+
b4+
y4+
y5+
b5+
y10++
y6+
b7+
y7+
y8+
b10+
y10+
m/z
PGNVKNSPGNK+XL_L
y4++
y7++
y8++
b6+
b9+
y9+
GYIVIEDLWKQNFQK+XL_S
GYIVIEDLWKQNFQK+XL_L
